# Sour Taste SNP *KCNJ2*-rs236514 and Differences in Nutrient Intakes and Metabolic Health Markers in the Elderly

**DOI:** 10.3389/fnut.2021.701588

**Published:** 2021-08-17

**Authors:** Celeste Ferraris, Alexandria Turner, Christopher J. Scarlett, Martin Veysey, Mark Lucock, Tamara Bucher, Emma L. Beckett

**Affiliations:** ^1^School of Environmental and Life Sciences, The University of Newcastle, Ourimbah, NSW, Australia; ^2^School of Medicine & Public Health, The University of Newcastle, Gosford, NSW, Australia; ^3^Hull York Medical School, University of Hull, Cottingham, United Kingdom; ^4^Priority Research Centre for Physical Activity and Nutrition, The University of Newcastle, Callaghan, NSW, Australia; ^5^Hunter Medical Research Institute, Newcastle, NSW, Australia

**Keywords:** sour, taste, genetics, KCNJ2, macronutrient, vitamin, mineral, metabolic

## Abstract

Single nucleotide polymorphisms (SNPs) in taste receptors influence dietary choices that contribute to health and quality of life. Individual differences in sour taste perception and preference have been linked to heritable genetics, yet the impact of sour taste receptor SNPs on sour taste is under-researched, and studies on sour taste SNP associations to diet and health are lacking. Therefore, this study explored the relationships between the sour taste SNP *KCNJ2*-rs236514 and estimated macronutrient, vitamin and mineral intakes, and markers of metabolic health. Associations were explored in 523 participants aged 65 years and older with data analysed using standard least squares and nominal logistic regression modelling with *post hoc* student's *t*-tests and Tukey's HSD. Associations were found between the presence of the *KCNJ2*-rs236514 variant allele (A) and lower intakes of energy, total fat, monounsaturated fat and saturated fat. The lower fat intakes were significant in female carriers of the variant allele (A), along with lower water intake. Lower retinol, riboflavin, folate, calcium and sodium intakes were found in the *KCNJ2*-A allele carriers. In females, the variant allele was associated with lower sodium intake before and after Bonferroni adjustment. Higher body mass index, waist and waist-to-hip ratio measures were found in males carrying the variant allele. Lower levels of liver function biomarkers were associated with the presence of the *KCNJ2*-A allele. Overall and in males, the variant's association to lower gamma-glutamyl transferase (GGT) levels remained significant after Bonferroni adjustments. These novel findings suggest the sour taste SNP, *KCNJ2*-rs236514, may be modifying macronutrient, vitamin and mineral intakes, and markers of metabolic health. Research on the extra-oral functions of this SNP may improve health outcomes for those with overweight, obesity and liver disease.

## Introduction

Diet is a key determinant of non-communicable health outcomes and quality of life (1, 2). This is becoming particularly important in the context of an ageing population. Eating preferences and dietary intake are influenced by individual differences in our perception and sensitivity to the five key tastes—bitter, sweet, umami, sour and salt (3–6). Genetic contributions to taste differences have been established in studies on variance in genes coding for taste receptors (6–10). Research on the consequential alterations to dietary patterns has primarily focused on the bitter taste genes (11, 12). The metabolic health implications of these taste-gene associated dietary choices has focused on bitter and sweet taste-related polymorphisms (13–15). However, the direct associations between many variants in taste genes, nutrient intake and biomarkers of health remain to be elucidated.

Sour taste can evoke both pleasant and aversive responses (16). Aversive responses may lead to the avoidance of healthy foods such as citrus fruits, berries, and fermented foods (17). Differences in sensitivity to sour taste have been found between the sexes. Women have higher perception thresholds and prefer sour more than men (18, 19), and neural responses to sour are stronger in women (20). The influence of genetics on variations in taste thresholds for the sourness of citric acid has been demonstrated in twin studies (9). Preference for sour has been more strongly correlated to genetic factors than environmental factors (10). However, the genetic variance in receptors responsible for the detection of sour compounds remains under-researched.

While several sour taste receptors have been proposed (21, 22), downstream sour signalling through an inwardly rectifying potassium channel appears to modulate the strength of transduction (23). *KCNJ2* (Potassium Inwardly Rectifying Channel Subfamily J Member 2) is a protein-coding gene directly linked to the magnitude of the inward potassium current and hence strength of sour transduction (24, 25). Alterations to sour taste have been linked to a single nucleotide polymorphism (SNP) in the *KCNJ2* gene (5). Carriers of the *KCNJ2*-rs236514 variant allele (A) have been shown to have a higher preference for sour, an association that was maintained after correction for multiple testing (5). In our study of the associations between the presence of this sour SNP and mild cognitive impairment, we reported that there was no association between three indices of diet quality and the presence of the variant (A) allele (26). However, diet quality indices provide only a high-level view of nutritional sufficiency, and the relationship between the *KCNJ2*-rs236514 polymorphism on nutrient intake has not been investigated.

Therefore, this study aimed to explore the associations between *KCNJ2*-rs236514, estimated habitual macronutrient, vitamin and mineral intakes, and biomarkers of metabolic health in an elderly cohort. While taste thresholds for sour and all other tastes have been shown to increase in ageing populations (27), there is an absence of research on how nutrient intake and biomarkers of health are affected. Furthermore, the impact of variance in genes coding for sour taste on diet and biomarkers of health in older populations has not been studied.

## Materials and Methods

### Subjects

This secondary analysis utilised the Retirement Health and Lifestyle Study (RHLS) cross-sectional cohort of adults aged 65 years and older who were living independently in the Central Coast area of NSW, Australia (28–31). Participants were required to have completed a valid food frequency questionnaire (FFQ) and provided blood samples to enable genotyping of *KCNJ2*-rs236514 for eligibility to this study. Complete data sets for 523 participants were available for the analyses. Written informed consent was obtained from participants and the University of Newcastle Human Research Ethics Committee provided ethics approval (Reference No. H-2008-0431) (29).

### Demographics and Anthropometrics

Demographic data (age, sex, income, education, history of smoking) were collated through interviewer-administered questionnaires (30, 32, 33). Body dimension (hip circumference, waist circumference, and height) and weight measurements were collected adhering to the standards of the International Society for the Advancement of Kinanthropometry (ISAK) (34). Body mass index (BMI) and waist to hip ratios (WHR) were calculated using standard equations (34).

### Blood Collection and Analyses

After fasting, whole blood was collected by a trained nurse, into EDTA-lined tubes and stored at −20°C (35). The Hunter Area Pathology Service analysed the blood samples to obtain the liver function, glucose, and lipid biomarker data (35). The biomarkers of liver function were gamma-glutamyl transferase (GGT), alkaline phosphatase (ALP), alanine aminotransferase (ALT), aspartate aminotransferase (AST), total protein, albumin, calcium globulin ratio (Cal/Glob), and total bilirubin. Along with blood glucose levels, glycosylated haemoglobin (HbA_1c_) was measured. The lipid biomarkers assessed were triglycerides (TG), low-density lipoprotein (LDL), high-density lipoprotein (HDL), total cholesterol (TC), and the ratio of TC to HDL.

### Genotyping

DNA was isolated from peripheral blood cells using QIAGEN QIAmp DNA mini kits (30, 36). The *KCNJ2*-rs236514 SNP was assessed via allelic discrimination using TaqMan^™^ assay (Applied Biosystems^™^, ThermoFisher Scientific, California, USA) and quantitative polymerase chain reaction (QuantStudio 7 Flex Real-Time PCR System) (37, 38). Manufacturers' protocols were followed.

### Dietary Assessment

Intakes of 225 food items were recorded by completion of a previously validated FFQ (39). Data were extracted for macronutrient, vitamin and mineral estimated habitual intakes with Foodworks^™^ (V.2.10.146) software (40). If participants' dietary reports were incomplete or energy intakes were <3,000 kJ/d or >30,000 kJ/d their FFQ was excluded.

### Blood Pressure Readings

Blood pressure (BP) measurements were taken from both arms by qualified clinical staff using an OMRON IA2 machine (32). Physical limitation preventing measurement, repetitive differences in systolic BP of >10 mmHg and diastolic BP of >6 mmHg, very high BP curtailing measurement and machine error were exclusion criteria (32). Following the World Health Organisation's guidelines, hypertensive was defined as recording systolic BP of ≥140 mmHg and diastolic BP of ≥90 mmHg (41). Additionally, those taking anti-hypertensive medications were classified as hypertensive (42).

### Statistical Analyses

The data analyses were undertaken using JMP (Pro V.14.2.0; SAS Institute Inc., Cary, NC, USA 27513). Continuous variable distributions (means, 95% confidence intervals and standard deviations) and categorical variable distributions (number and percentage of cohort) describe the cohort characteristics. Analysis of the polymorphism, *KCNJ2*-rs236514, occurred by presence or absence of the variant allele (A) and was reported as the number and percentage of the study cohort. Results were further stratified by sex. Analyses were repeated using genotypes to investigate potential allele dose dependent responses, where appropriate using ANOVA and Tukey's *post-hoc* test to compare means between groups.

Statistical significance of continuous variables was examined through standard least squares regression analyses and for categorical variables through nominal logistic regression analyses (χ^2^, *p*-values) with *post hoc* student's *t*-test (two categories) and Tukey's HSD (three categories). *p*-values are presented to one significant number and threshold *p*-values of <0.05 were considered statistically significant. The Bonferroni method was applied to correct for multiple testing and the alternative adjusted thresholds are presented (43). Where appropriate, results were adjusted for potential confounding factors such as age, sex, education, income, smoking status, BMI, and energy intake. Due to the small number of participants that reported smoking, current smokers and ex-smokers were collapsed to “history of smoking.”

## Results

### Participant Characteristics

The average age of the 523 participants was 77.5 (SD ± 6.7) years and did not differ by sex (Supplementary Table 1). The cohort was 54.5% female (Supplementary Table 2). Most participants earned between $20,000–$60,000/year; however, distributions of income categories varied by sex with men reporting earning more than women (*p* < 0.0001; Supplementary Table 2). Men were more likely to be educated at TAFE (Technical and Further Education) level or higher (75.6 vs. 60.2%, *p* = 0.001) and to have a history of smoking (66.4 vs. 35.1%, *p* < 0.0001; Supplementary Table 2). Weight, waist, and hip measures were normally distributed (Supplementary Table 1). Men were taller, weighed more, and had higher waist and WHR measures (*p* < 0.0001; Supplementary Table 1). The mean BMI was 28.6 kg/m^2^ (SD ± 4.8) and did not vary by sex (Supplementary Table 1).

### Genotype Distributions

The *KCNJ2*-rs236514 variant allele (A) had a frequency of 0.56. The proportion of participants carrying the *KCNJ2*-A allele (AA or AG genotypes) was 81.3% and there was no statistically significant difference by sex (Supplementary Table 3).

### Relationships Between *KCNJ2*-rs236514 and Confounding Variables

The presence of the *KCNJ2*-A allele did not vary by age, sex, income, education, history of smoking or BMI in the total cohort (Supplementary Tables 4, 5). The mean age of female *KCNJ2*-A allele carriers was older than non-carriers (78.1 vs. 75.9 years, *p* = 0.04) and mean BMI was higher in male *KCNJ2*-A allele carriers (29.0 vs. 26.8 kg/m^2^, *p* = 0.003; Supplementary Table 4).

### Relationships Between *KCNJ2*-rs236514 and Estimated Energy and Macronutrient Intakes

The *KCNJ2*-rs236514 variant allele (A) was associated with lower mean intakes of energy, TF, MUFA and SF (*p* range = 0.02–0.04; Table 1). Relationships for TF, MUFA and SF remained significant after adjusting for age and sex (*p* range = 0.02–0.03). However, relationships were not significant in the fully adjusted model (age, sex, income, education, and BMI). Relationships were not significant when Bonferroni corrections were applied for multiple testing (adjusted *p*-threshold ≤0.004).

**Table 1:** Estimated energy and macronutrient intakes vary by the presence of the *KCNJ2*-A allele in unadjusted and adjusted models.

**Macronutrient**	**Unadjusted**			**Model 1**			**Model 2**		
	***Mean (95% CI)***		***p***	***Mean (95% CI)***		***p***	***Mean (95% CI)***		***p***
	**A allele absent**	**A allele present**		**A allele absent**	**A allele present**		**A allele absent**	**A allele present**	
Energy kJ/d	8720.5 (8177.6–9263.5)	8102.2 (7841.4–8363.0)	**0.04**	8699.0 (8160.1–9237.8)	8147.5 (7888.0–8407.0)	0.07	8977.2 (8352.6–9601.8)	8520.2 (8113.1–8927.2)	0.2
Carbohydrate g/d	223.3 (207.2–239.3)	214.2 (206.5–222.0)	0.3	223.6 (207.6–239.7)	215.1 (207.3–222.8)	0.4	232.9 (214.6–251.2)	225.3 (213.3–237.2)	0.4
Fibre g/d	32.3 (29.8–34.8)	30.9 (29.8–32.1)	0.3	32.2 (29.7–34.7)	31.0 (29.8–32.2)	0.4	33.9 (31.0–36.7)	32.6 (30.7–34.4)	0.4
Starch g/d	105.0 (97.1–112.9)	101.2 (97.1–105.2)	0.4	104.4 (96.4–112.6)	102.3 (98.4–106.2)	0.6	105.2 (95.8–114.7)	103.5 (97.4–109.7)	0.7
Sugars g/d	116.3 (106.0–126.5)	110.9 (105.8–115.7)	0.4	117.1 (106.9–127.3)	110.5 (105.6–115.5)	0.3	125.4 (113.8–136.9)	119.3 (111.7–126.8)	0.3
Protein g/d	95.4 (89.4–101.5)	90.2 (87.2–93.2)	0.1	95.0 (88.8–101.2)	90.6 (87.6–93.5)	0.2	94.7 (87.4–102.0)	92.1 (87.3–96.8)	0.5
Total Fat g/d	75.7 (69.7–81.8)	67.4 (64.4–70.3)	**0.02**	75.4 (69.3–81.5)	67.6 (65.7–70.5)	**0.02**	78.5 (71.3–85.7)	71.7 (67.0–76.3)	0.06
MUFA g/d	29.9 (27.3–32.6)	26.3 (25.0–27.3)	**0.02**	29.7 (27.1–32.3)	26.4 (25.2–27.7)	**0.03**	31.0 (27.9–34.0)	28.2 (26.2–30.2)	0.07
PUFA g/d	12.5 (10.9–14.1)	11.2 (10.5–12.0)	0.2	12.4 (10.8–14.0)	11.2 (10.5–12.0)	0.2	13.1 (11.2–15.0)	12.1 (10.9–13.3)	0.3
SF g/d	25.9 (23.7–28.2)	23.2 (22.1–24.2)	**0.03**	25.9 (23.7–28.1)	23.2 (22.2–24.3)	**0.03**	27.0 (24.4–29.6)	24.5 (22.9–26.3)	0.07
Cholesterol g/d	258.7 (236.9–280.5)	242.1 (231.6–252.5)	0.2	257.5 (236.7–279.3)	243.0 (232.5–254.5)	0.2	256.8 (231.3–282.4)	246.8 (230.1–263.4)	0.4
Alcohol g/d	10.3 (7.8–12.9)	8.3 (7.1–9.5)	0.2	10.1 (7.7–12.5)	8.8 (7.7–10.0)	0.3	10.1 (7.3–12.8)	9.3 (7.5–11.1)	0.6
Water g/d	3149.1 (2911.8–3386.5)	2909.9 (2806.8–3013.0)	0.05	3136.1 (2917.2–3355.0)	2916.3 (2810.9–3021.7)	0.08	3227.3 (2976.4–3478.2)	3015.7 (2852.1–3179.2)	0.1

*Model 1, adjusted for age and sex; Model 2, adjusted for age, sex, income, education, smoking, and BMI*.

*CI, confidence interval; MUFA, monounsaturated fat; PUFA, polyunsaturated fat; SF, saturated fat; Bonferroni adjusted p-threshold ≤ 0.004. Bold values indicates statistically significant*.

Differences in energy and macronutrient intake distributions were found between the sexes. Men consumed more energy, carbohydrate, starch, protein, and alcohol than women (*p* range <0.0001–0.04; Supplementary Table 6). Therefore, the analyses were stratified by sex (Supplementary Table 7A). In unadjusted, and age-adjusted models, TF and MUFA intake were lower in females who carried the *KCNJ2*-A allele (*p* range = 0.02–0.03), but these relationships were not seen in males. Lower SF intake was found in female *KCNJ2*-A allele carriers in the age-adjusted model only (*p* = 0.04). Additionally, females who carried the *KCNJ2*-A allele had lower daily water intakes across all models (*p* range = 0.01–0.04). Relationships were not significant when Bonferroni corrections were applied for multiple testing (adjusted *p*-threshold ≤0.004).

### Relationships Between *KCNJ2*-rs236514 and Estimated Dietary Vitamin Intakes

Dietary retinol, riboflavin and folate intakes were lower in those carrying the *KCNJ2*-A allele, in the unadjusted and adjusted models (*p* range = 0.005–0.02; Table 2). Women consumed less dietary thiamine, niacin, niacin equivalents, and folate and greater amounts of beta-carotene than men (Supplementary Table 8). Therefore, analyses were stratified by sex (Supplementary Table 7B). In women, the presence of the *KCNJ2*-A allele was associated with lower folate intakes in the unadjusted and age-adjusted models (*p* = 0.02). In men, *KCNJ2*-A allele presence was associated with lower intakes of retinol, thiamine, and riboflavin in all models (*p* range = 0.008–0.03). Relationships were not significant when Bonferroni corrections were applied for multiple testing (adjusted *p*-threshold ≤0.004).

**Table 2:** Estimated dietary vitamin intakes vary by the presence of the *KCNJ2*-A allele in unadjusted and adjusted models.

	**Unadjusted**			**Model 1**			**Model 2**		
**Vitamin**	***Mean (95% CI)***		***p***	***Mean (95% CI)***		***p***	***Mean (95% CI)***		***p***
	**A allele absent**	**A allele present**		**A allele absent**	**A allele present**		**A allele absent**	**A allele present**	
Total A eq. (μg/d)	1906.5 (1642.7–2170.3)	1724.1 (1597.5–1850.8)	0.2	1922.4 (1658.5–2186.4)	1712.3 (1585.1–1839.4)	0.2	2057.5 (1744.9–2370.0)	1878.2 (1674.5–2081.9)	0.3
Retinol (μg/d)	498.3 (358.4–638.3)	284.3 (217.1–351.5)	**0.007**	503.6 (363.3–643.9)	287.6 (220.0–355.1)	**0.007**	502.1 (327.9–676.2)	281.4 (167.9–394.9)	**0.01**
Beta-carotene (μg/d)	8430.3 (7141.0–9719.7)	8619.2 (8000.109238.3)	0.8	8494.5 (7208.6–9780.4)	8528.6 (7909.4–9147.8)	1.0	9313.0 (7838.0–10788.0)	9560.5 (8599.2–10521.7)	0.7
Thiamine (mg/d)	2.2 (2.0–2.4)	1.9 (1.9–2.0)	0.05	2.2 (2.0–2.4)	2.0 (1.9–2.1)	0.06	2.2 (1.9–2.4)	2.0 (1.8–2.1)	0.1
Riboflavin (mg/d)	2.7 (2.5–2.9)	2.4 (2.2–2.5)	**0.007**	2.7 (2.5–2.9)	2.3 (2.2–2.5)	**0.005**	2.7 (2.4–3.0)	2.4 (2.2–2.6)	**0.02**
Niacin (mg/d)	26.7 (24.9–28.5)	24.7 (23.8–25.6)	0.05	26.6 (24.8–28.4)	24.9 (24.0–25.8)	0.1	26.7 (24.6–28.8)	25.4 (24.0–26.7)	0.2
Niacin eq. (mg/d)	45.8 (42.8–48.8)	42.8 (41.4–44.3)	0.08	45.6 (42.6–48.5)	43.1 (41.7–44.5)	0.1	45.7 (42.3–49.1)	43.9 (41.7–46.2)	0.3
Vitamin B6 (mg/d)	2.9 (1.2–4.5)	3.4 (2.6–4.2)	0.6	2.9 (1.3–4.6)	3.4 (2.6–4.2)	0.6	3.8 (2.1–5.5)	4.2 (3.1–5.3)	0.7
Folate (μg/d)	569.5 (529.4–609.5)	506.6 (487.3–525.8)	**0**.**006**	570.0 (530.3–609.7)	510.0 (490.8–529.1)	**0.008**	569.2 (522.7–615.6)	511.7 (481.4–542.0)	**0.02**
Vitamin B12 (μg/d)	5.3 (4.8–5.9)	5.2 (4.9–5.4)	0.6	5.3 (4.7–5.9)	5.2 4.9 (5.5)	0.7	5.2 (4.6–5.8)	5.2 (4.8–5.6)	1.0
Vitamin C (mg/d)	218.0 (193.3–242.7)	210.2 (198.3–222.0)	0.6	218.8 (194.0–243.5)	209.8 (197.9–221.8)	0.5	238.1 (210.0–266.2)	225.0 (206.7–243.3)	0.4
Vitamin D (μg/d)	2.6 (2.2–2.9)	2.5 (2.4–2.7)	0.8	2.6 (2.2–2.9)	2.5 (2.4–2.7)	0.8	2.6 (2.3–3.0)	2.6 (2.4–2.8)	0.8
Vitamin E (mg/d)	10.0 (9.1–10.9)	9.3 (8.9–9.7)	0.1	10.0 (9.1–10.8)	9.3 (8.9–9.7)	0.2	10.6 (9.6–11.5)	10.0 (9.4–10.6)	0.2

*eq., equivalents; Model 1, adjusted for age and sex; Model 2, adjusted for age, sex, education, income, smoking and BMI*.

*CI, confidence interval; Bonferroni adjusted p-threshold ≤0.004. Bold values indicates statistically significant*.

### Relationships Between *KCNJ2*-rs236514 and Estimated Dietary Mineral Intakes

In all models, dietary calcium and sodium intakes were lower in those carrying the *KCNJ2*-A allele (*p* range = 0.01–0.04; Table 3). Relationships were not significant when Bonferroni corrections were applied for multiple testing (adjusted *p*-threshold ≤0.006). Differences in mineral intakes were found between the sexes, with men consuming more dietary iron, magnesium, sodium, and zinc (Supplementary Table 9). Therefore, the analyses were stratified by sex (Supplementary Table 7C). In women, the presence of the *KCNJ2*-A allele was associated with lower sodium intakes in all models (*p* range = 0.0006–0.007). Relationships were significant when Bonferroni corrections were applied for multiple testing in the unadjusted and age-adjusted models (adjusted *p*-threshold ≤0.006). In men, the presence of the *KCNJ2*-A allele was associated with lower calcium intakes in the fully adjusted model only (*p* = 0.04). This relationship was not significant when Bonferroni corrections were applied for multiple testing (adjusted *p*-threshold ≤0.006).

**Table 3:** Estimated dietary mineral intakes vary by the presence of the *KCNJ2*-A allele in unadjusted and adjusted models.

	**Unadjusted**			**Model 1**			**Model 2**		
**Minerals**	***Mean (95% CI)***		***p***	***Mean (95% CI)***		***p***	***Mean (95% CI)***		***p***
	**A allele absent**	**A allele present**		**A allele absent**	**A allele present**		**A allele absent**	**A allele present**	
Calcium (mg/d)	1036.7 (949.9–1123.4)	932.7 (891.0–974.3)	**0.03**	1041.3 (954.6–1128.0)	927.9 (886.1–969.6)	**0.02**	1078.2 (974.8–1181.5)	970.9 (903.6–1038.3)	**0.04**
Copper (mg/d)	2.4 (2.2–2.6)	2.3 (2.2–2.4)	0.2	2.4 (2.2–2.6)	2.3 (2.2–2.4)	0.2	2.6 (2.4–2.8)	2.5 (2.3–2.6)	0.2
Iron (mg/d)	15.4 (14.3–16.4)	14.3 (13.8–14.8)	0.07	15.3 (14.3–16.4)	14.4 (13.9–14.9)	0.1	15.6 (14.4–16.8)	14.8 (14.0–15.6)	0.2
Magnesium (mg/d)	416.2 (387.3–445.0)	391.3 (377.4–405.1)	0.1	414.7 (385.9–443.5)	392.8 (379.0–406.7)	0.2	436.9 (403.4–470.4)	418.8 (397.0–440.6)	0.3
Potassium (mg/d)	4419.6 (4112.2–4727.0)	4144.5 (3997.2–4291.9)	0.1	4418.7 (4110.1–4727.3)	4150.2 (4001.9–4298.5)	0.1	4627.6 (4272.0–4983.1)	4399.9 (4168.7–4631.1)	0.2
Selenium (mg/d)	178.4 (144.5–212.3)	169.9 (153.6–186.1)	0.7	176.7 (142.7–210.7)	170.0 (153.7–186.4)	0.7	182.4 (141.4–223.5)	182.4 (155.6–209.1)	1.0
Sodium (mg/d)	2224.1 (2067.2–2381.1)	1994.3 (1918.9–2069.7)	**0.01**	2209.7 (2057.1–2362.4)	2017.5 (1944.0–2091.0)	**0.03**	2225.5 (2044.6–2406.4)	2040.6 (1922.7–2158.5)	**0.04**
Zinc (mg/d)	13.4 (12.4–14.3)	12.6 (12.2–13.1)	0.2	13.3 (12.4–14.3)	12.7 (12.2–13.1)	0.2	13.6 (12.5–14.7)	13.0 (12.3–13.8)	0.3

*Model 1, adjusted for age and sex; Model 2, adjusted for age, sex, education, income, smoking and BMI*.

*CI, confidence interval; Bonferroni adjusted p-threshold ≤0.006. Bold values indicates statistically significant*.

### Relationships Between *KCNJ2*-rs236514 and Body Composition Markers

The presence of the *KCNJ2*-A allele was not associated with the body composition markers in adjusted or unadjusted models (Table 4). However, male participants had higher mean weight, waist, and WHR distributions than females (*p* < 0.0001; Supplementary Table 1). Therefore, the analyses were stratified by sex (Supplementary Table 7D). The presence of the *KCNJ2*-A allele in males was associated with higher mean scores for BMI, waist and WHR in the unadjusted, age adjusted, fully adjusted, and in an additional fully adjusted model inclusive of energy intake as a variable (*p* range = 0.0007–0.004). Except for waist and WHR in the unadjusted models, these relationships were significant when Bonferroni corrections were applied for multiple testing (adjusted *p*-threshold ≤0.01). In females, the presence of the *KCNJ2*-A allele was associated with lower WHR scores after adjusting for age, education, income, and smoking (*p* = 0.03); and age, education, income, smoking and energy intake (*p* = 0.04). These relationships were not significant when Bonferroni corrections were applied for multiple testing (adjusted *p*-threshold ≤0.01).

**Table 4:** Body composition markers do not vary by the presence of the *KCNJ2*-A allele in unadjusted and adjusted models.

**Body composition marker**	**Unadjusted**			**Model 1**			**Model 2**			**Model 3**		
	***Mean (95% CI)***		***p***	***Mean (95% CI)***		***p***	***Mean (95% CI)***		***p***	***Mean (95% CI)***		***p***
	**A allele absent**	**A allele present**		**A allele absent**	**A allele present**		**A allele absent**	**A allele present**		**A allele absent**	**A allele present**	
Weight (kg)	76.3 (73.2–79.4)	76.3 (74.8–77.8)	1.0	75.6 (72.9–78.2)	77.2 (75.9–78.5)	0.3	75.6 (72.9–78.2)	77.2 (75.9–78.5)	0.3	73.9 (71.0–76.9)	75.7 (73.8–77.6)	0.2
BMI (kg/m^2^)	27.9 (27.0–29.0)	28.7 (28.2–29.2)	0.2	27.8 (26.8–28.8)	28.7 (28.3–29.2)	0.08	27.8 (26.8–28.8)	28.7 (28.3–29.2)	0.08	27.3 (26.2–28.3)	28.3 (27.6–29.0)	0.07
Waist (cm)	97.7 (95.9–100.5)	99.2 (97.9–100.5)	0.3	97.4 (94.9–99.8)	99.9 (98.7–101.1)	0.07	97.4 (94.9–99.8)	99.9 (98.7–101.1)	0.07	96.3 (93.6–99.0)	98.9 (97.1–100.6)	0.06
Hip (cm)	108.3 (106.3–110.4)	109.4 (108.5–110.4)	0.3	108.1 (106.0–110.1)	109.4 (108.5–110.4)	0.3	108.1 (106.0–110.1)	109.4 (108.5–110.4)	0.3	107.4 (105.1–109.7)	108.7 (107.2–110.2)	0.3
WHR	0.91 (0.89–0.92)	0.91 (0.89–0.91)	1.0	0.91 (0.89–0.92)	0.91 (0.90–0.92)	0.4	0.91 (0.89–0.92)	0.91 (0.90–0.92)	0.4	0.90 (0.89–0.92)	0.91 (0.90–0.92)	0.3

*Model 1, adjusted for age and sex; Model 2, adjusted for age, sex, education, income and smoking; Model 3, adjusted for age, sex, education, income, smoking and energy intake*.

*CI, confidence interval; BMI, Body Mass Index; WHR, waist to hip ratio*.

### Relationships Between *KCNJ2*-rs236514 and Liver Function Biomarkers

The presence of the *KCNJ2*-A allele was associated with lower levels of blood GGT and AST in all models (*p* range = 0.0002–0.01; Table 5). Lower blood albumin levels were associated with the presence of the *KCNJ2*-A allele in the unadjusted and fully adjusted models (*p* = 0.03; Table 5). Relationships to lower GGT levels in all models were significant when Bonferroni corrections were applied for multiple testing (adjusted *p*-threshold ≤0.006).

**Table 5:** Liver function biomarkers vary by the presence of the *KCNJ2*-A allele in unadjusted and adjusted models.

**Liver function biomarkers**	**Unadjusted**			**Model 1**			**Model 2**		
	***Mean (95% CI)***		***p***	***Mean (95% CI)***		***p***	***Mean (95% CI)***		***p***
	**A allele absent**	**A allele present**		**A allele absent**	**A allele present**		**A allele absent**	**A allele present**	
GGT (U/L)	57.2 (47.2–67.2)	36.5 (31.7–41.3)	**0.0003**	57.8 (47.9–67.7)	37.1 (32.3–41.9)	**0.0002**	55.7 (43.7–67.6)	36.2 (28.4–44.0)	**0.001**
ALP (U/L)	78.5 (71.7–85.3)	76.1 (72.8–79.4)	0.5	79.1 (72.2–85.9)	76.1 (72.8–79.4)	0.5	79.2 (70.9–87.6)	76.4 (71.0–81.8)	0.5
ALT (U/L)	24.5 (22.4–26.6)	22.3 (21.2–23.3)	0.06	24.2 (22.1–26.2)	22.5 (21.5–23.5)	0.1	24.8 (22.5–27.0)	23.3 (21.8–24.8)	0.2
AST (U/L)	21.6 (20.0–23.1)	19.3 (18.6–20.1)	**0.01**	21.6 (20.1–23.2)	19.3 (18.6–20.1)	**0.009**	22.4 (20.6–24.2)	20.1 (18.9–21.2)	**0.01**
T. Protein (g/L)	76.8 (75.7–77.6)	76.3 (75.8–76.7)	0.4	76.7 (75.8–77.6)	76.3 (75.9–76.8)	0.5	76.3 (75.2–77.3)	75.8 (75.1–76.5)	0.4
Albumin (g/L)	39.5 (38.9–40.0)	38.8 (38.5–39.1)	**0.03**	39.4 (38.9–40.0)	38.8 (38.6–39.1)	0.05	39.3 (38.7–39.9)	38.6 (38.2–39.0)	**0.03**
Cal/Glob	37.3 (36.3–38.2)	37.5 (37.0–37.9)	0.7	37.3 (36.4–38.2)	37.5 (37.1–37.9)	0.7	37.0 (35.9–38.0)	37.1 (36.4–37.8)	0.8
Bilirubin (μmol/L)	11.7 (10.8–12.6)	10.6 (10.2–11.1)	0.05	11.7 (10.8–12.6)	10.8 (10.3–11.2)	0.05	11.8 (10.8–12.8)	10.8 (10.2–11.5)	0.05

*Model 1, adjusted for age and sex; Model 2, adjusted for age, sex, education, income, smoking and BMI*.

*CI, confidence interval; GGT, gamma glutamyltransferase; ALP, alkaline phosphatase; ALT, alanine aminotransferase; AST, aspartate aminotransferase; Cal/Glob, calcium globulin ratio; Bonferroni adjusted p-threshold ≤0.006. Bold values indicates statistically significant*.

Higher blood GGT, ALT, total protein, albumin, and bilirubin levels were found in male participants (*p* range <0.0001–0.006; Supplementary Table 10), therefore the analyses were stratified by sex (Supplementary Table 7E). In males, the presence of the *KCNJ2*-A allele was associated with lower GGT and ALT, across all models (*p* range = 0.0002–0.001). The variant allele (A) was also associated with lower albumin and bilirubin levels in males, in all models (*p* range = 0.03–0.04). In women, the presence of the *KCNJ2*-A allele was associated with lower blood AST in the fully adjusted model (*p* = 0.04). Relationships to lower GGT levels in male *KCNJ2*-A allele carriers were significant in all models when Bonferroni corrections were applied for multiple testing (adjusted *p*-threshold ≤0.006).

### Relationships Between *KCNJ2*-rs236514 and Blood Glucose Levels

A statistically significant association was found between lower fasting blood glucose levels and the presence of the *KCNJ2*-A allele in the fully adjusted model (*p* = 0.02; Table 6). Fasting blood glucose levels were significantly higher in men than in women (*p* < 0.0001; Supplementary Table 11), therefore the analyses were stratified by sex (Supplementary Table 7F). The presence of the *KCNJ2*-A allele in males was associated with lower fasting blood glucose in the fully adjusted model only (*p* = 0.005).

**Table 6:** Blood glucose measures vary by the presence of the *KCNJ2*-A allele in the fully adjusted model.

**Blood glucose measures**	**Unadjusted**			**Model 1**			**Model 2**		
	***Mean (95% CI)***		***p***	***Mean (95% CI)***		***p***	***Mean (95% CI)***		***p***
	**A allele absent**	**A allele present**		**A allele absent**	**A allele present**		**A allele absent**	**A allele present**	
Glucose^*^ (mmol/L)	5.8 (5.5–6.0)	5.6 (5.5–5.7)	0.09	5.7 (5.5–5.9)	5.6 (5.5–5.7)	0.2	5.7 (5.5–6.0)	5.5 (5.3–5.6)	**0.02**
HbA_1c_ (%)	5.9 (5.8–6.0)	5.9 (5.9–6.0)	0.8	5.9 (5.8–6.0)	5.9 (5.9–6.0)	0.9	5.9 (5.8–6.0)	5.9 (5.8–6.0)	0.6

*Model 1, adjusted for age and sex; Model 2, adjusted for age, sex, education, income, smoking and BMI*.

*CI, confidence interval*.

* *Fasting; HbA_1c_, glycosylated haemoglobin. Bold values indicates statistically significant*.

### Relationships Between *KCNJ2*-rs236514 and Blood Lipid Levels

There were no associations between the blood lipids and the presence of the *KCNJ2*-A allele (Table 7). However, female participants had higher blood levels of total cholesterol, LDL and HDL, while males had a higher mean total cholesterol to HDL ratio (*p* range <0.0001–0.04; Supplementary Table 12). Therefore, the analyses were stratified by sex (Supplementary Table 7G). The presence of the *KCNJ2*-A allele in females was associated with higher LDL levels, however only in the age-adjusted model (*p* = 0.04). Relationships were not significant when Bonferroni corrections were applied for multiple testing (adjusted *p*-threshold ≤0.01).

**Table 7:** Blood lipid measures do not vary by the presence of the *KCNJ2*-A allele in unadjusted and adjusted models.

**Blood lipid measures**	**Unadjusted**			**Model 1**			**Model 2**		
	***Mean (95% CI)***		***p***	***Mean (95% CI)***		***p***	***Mean (95% CI)***		***p***
	**A allele absent**	**A allele present**		**A allele absent**	**A allele present**		**A allele absent**	**A allele present**	
TG (mmol/L)	1.4 (1.2–1.5)	1.4 (1.3–1.4)	0.9	1.4 (1.2–1.5)	1.4 (1.3–1.4)	1.0	1.3 (1.2–1.5)	1.3 (1.2–1.4)	0.9
LDL (mmol/L)	2.4 (2.2–2.6)	2.5 (2.4–2.6)	0.3	2.4 (2.2–2.6)	2.5 (2.4–2.6)	0.3	2.3 (2.1–2.5)	2.4 (2.3–2.6)	0.4
HDL (mmol/L)	1.5 (1.4–1.6)	1.5 (1.4–1.5)	0.2	1.5 (1.4–1.6)	1.4 (1.4–1.5)	0.1	1.6 (1.5–1.6)	1.5 (1.4–1.6)	0.3
TC (mmol/L)	4.5 (4.3–4.8)	4.6 (4.5–4.7)	0.6	4.5 (4.3–4.7)	4.6 (4.5–4.7)	0.7	4.5 (4.3–4.7)	4.5 (4.4–4.7)	0.6
TC/HDL	3.2 (3.0–3.5)	3.4 (3.3–3.5)	0.3	3.2 (3.0–3.4)	3.4 (3.3–3.5)	0.2	3.1 (2.9–3.4)	3.2 (3.1–3.4)	0.4

*Model 1, adjusted for age and sex; Model 2, adjusted for age, sex, education, income, smoking and BMI*.

*CI, confidence interval; TG, triglycerides, LDL, low density lipoprotein, HDL, high density lipoprotein; TC, total cholesterol; Bonferroni adjusted p-threshold ≤0.01*.

### Relationships Between *KCNJ2*-rs236514 and Hypertension

The presence of the *KCNJ2*-A allele was not associated with hypertension in the total cohort, in unadjusted and adjusted models (Table 8). There were no differences between the sexes in the distribution analyses (Supplementary Table 13) and no associations found when results were stratified by sex (Supplementary Table 7H).

**Table 8:** Hypertension status does not vary by presence of the *KCNJ2*-A allele in unadjusted and adjusted models.

	**Unadjusted**		**Model 1**		**Model 2**	
	**χ** ^**2**^ **(*p*-value)**	**OR (95% CI)**	**χ** ^**2**^ **(*p*-value)**	**OR (95% CI)**	**χ** ^**2**^ **(*p*-value)**	**OR (95% CI)**
	**A allele present**					
**Hypertensive**	0.1	1.1	0.04	1.1	0.02	1.04
	(0.8)	(0.6–1.7)	(0.8)	(0.6–1.7)	(0.9)	(0.6–1.8)

*Hypertensive, systolic ≥140 mmHg and diastolic ≥90 mmH and/or on anti-hypertensive medication; Model 1, adjusted for age and sex; Model 2, adjusted for age, sex, income, education, smoking and BMI*.

*OR, odds ratio; CI, confidence interval*.

### Analysis by Genotype

Analysis by *KCNJ2*-rs236514 genotype was repeated for the relationships with estimated energy, macronutrient, vitamin and mineral intakes, body composition markers, liver function biomarkers, blood glucose levels, blood lipid levels and hypertension for the complete cohort, including unadjusted and adjustment models as above. Sex stratified analyses were not conduct by genotype due to insufficient statistical power.

When analysed by genotype energy intake, TF, MUFA, SF, retinol, riboflavin, folate, sodium, and calcium intakes showed potential allele dose dependent patterns, with the highest means in those homozygous for the G allele, the lowest in those homozygous for the A allele, and intermediate levels in the heterozygotes, in all models (Supplementary Table 14). However, there were only statistically significant differences between the homozygous groups for TF and MUFA, in the unadjusted model (Supplementary Table 14), and for retinol and folate in all models, and for riboflavin and sodium in the unadjusted and the age and sex adjusted models (Supplementary Table 14).

None of the body composition markers showed clear patterns of variance by genotype (Supplementary Table 15). When analysed by genotype GGT, AST, and fasting glucose showed potential allele dose dependent patterns, with the highest means in those homozygous for the G allele, the lowest in those homozygous for the A allele, and intermediate levels in the heterozygotes, in all models (Supplementary Table 16). However, there were only statistically significant differences between the homozygous groups for AST; those homozygous for the G allele and A allele carrying genotype groups for GGT, in all models; and between those homozygous for the G allele and A allele carrying genotype groups for fasting glucose in the fully adjusted model (Supplementary Table 16).

## Discussion

This study is the first to explore relationships between KCNJ2 genetic variation and measures of nutrient intake, body composition, and health-related biomarkers. Although the cross-sectional design has limitations, demonstrating these correlations in a convenient sample is a necessary first step to addressing the research gaps in this field. The results indicate that carriage of the *KCNJ*2-rs236514 variant allele (A) is related to differences in fat and water intake, estimated intake of various micronutrients, body composition, blood glucose levels and blood biomarkers of liver health in an elderly cohort, and that these relationships vary by sex. At a time when incidences of metabolic-related diseases are rapidly increasing worldwide (44), the results provide new understandings of possible drivers and important avenues for further research.

As a 3'-UTR polymorphism, the *KCNJ2*-rs236514 variant may alter protein expression and stability, rather than directly modulating the protein structure or function (45). While the ion channel coded by the *KCNJ2* gene increases magnitude of sour taste (24), there is uncertainty around the role of the *KCNJ2*-rs236514 variant. Prior research has shown *KCNJ2*-A allele carriers like sour more than non-carriers (5). Reduced liking of tastants has been demonstrated in the presence of taste receptor SNPs that increase intensity of taste perception (46, 47). Therefore, the variant may be reducing the magnitude of sour taste transduction. Due to the novel nature of these findings, this hypothesis and related research form the framework for discussion.

While the identified relationship between fat intake and sour taste genotype *KCNJ2*-rs236514 may seem counterintuitive, it is established that sour perception is suppressed by fats and vice-versa (48–52). Here, estimated consumption of TF, MUFA and SF were significantly lower amongst *KCNJ2*-A allele carriers, particularly in females. If the variant is reducing the magnitude of sour taste transduction, there may be an increase in liking for sour. As a result, lower consumption of dietary fat may be required to moderate the sourness of foods. This potential sex dimorphism is congruent with previous research showing women find sour more intense and are more sensitive to sourness (18, 19, 53). As female *KCNJ2*-A allele carriers consumed less fat, the hypothesis is supported. Further research is required to substantiate these theories and the bases from which they are made.

The finding in this study that female *KCNJ2*-A allele carriers consumed significantly less water is supported by previous research on sour taste receptors. The *PKD2LI* acid-sensing pathway was found to be activated by water, triggering appetitive drinking under thirst (54). Therefore, the reduced water intake of female *KCNJ2*-A allele carriers further supports the hypothesis that the SNP is reducing the degree of transduction. Additional research is required to explore this possibility and the mechanisms in play.

There were lower retinol, riboflavin, folate, calcium, and sodium intakes in *KCNJ2*-A allele carriers before and after adjustments, and differences by sex. Relationships between *KCNJ2* SNPs and micronutrient intakes have not previously been explored, therefore data are not available to contextualise these findings. However, as a fat-soluble vitamin (55), lower retinol intake may be explained by the lower intake of TF, MUFA and SF by those carrying the *KCNJ2*-A allele. These novel findings would benefit from further studies on *KCNJ2* variance, sour taste genetics more broadly, and vitamin and mineral intakes. Studies incorporating individual food intakes are required for more practical dietary understanding and application.

The body composition measures of BMI, waist and WHR were all significantly higher in male *KCNJ2*-A allele carriers in all adjustment models and after correction for multiple testing. The mean BMI places male *KCNJ2*-A allele carriers in the overweight category, and waist and WHR scores indicate an increased risk of metabolic complications (56). Energy intake did not modify the association suggesting an effect on body mass markers other than diet. The *KCNJ2* gene is expressed in high concentrations in human endocrine and brain tissues (57). Both areas play a role in metabolism hence extra-oral *KCNJ2* gene expression may be altering function in these tissues influencing body composition. As BMI, waist and WHR are indicators of obesity and obesity-related diseases (58), further research on the role of the KCNJ2 receptor would be valuable to fully elucidate it's gustatory and extra-oral functions.

The liver enzymes in the total cohort (GGT, AST) and in men (GGT, ALT, Albumin, Bilirubin) that were associated with the presence of the *KCNJ2*-A allele were significant at lower mean levels. Clinically, the mean GGT levels in non-*KCNJ2*-A allele carriers exceeded the reference ranges in the total cohort and men [reference range 5–50 U/L (59)]. However, the levels of GGT enzymes were ~50% lower in *KCNJ2*-A allele carriers than they were in non-carriers. Raised liver enzymes, particularly GGT, are significant risk factors for metabolic syndrome and type 2 diabetes (60, 61). Therefore, the lower GGT levels in variant allele (A) carriers may suggest a protective effect on metabolic health. Particularly considering the elevated metabolic-disease risk profile of male participants with the variant allele (A) in this study. Further exploration of these relationships in the context of presence or absence of liver disease, and in metabolic diseases are required. As the relationships between *KCNJ2*-A allele presence and the liver function biomarkers exist independently of all confounders and the KCNJ2 protein is moderately expressed in the human liver (57), the possible extra-oral functions of the receptor should be considered and investigated.

Fasting blood glucose levels were lower in *KCNJ2*-A allele carriers in the total cohort and in males, in the fully adjusted model. Clinically, fasting blood glucose levels are within healthy ranges in this cohort [reference range 3.0–6.0 mmol/L (62)]. Therefore, the statistical significance is not indicative of pathological significance. However, fasting blood glucose is positively correlated with obesity-related markers (63, 64). In this study fasting blood glucose was lower but BMI, waist and WHR scores were higher in male *KCNJ2*-A allele carriers. This supports the hypothesised extra-oral functions of the receptor, in line with its presence in endocrine and brain tissue. Furthermore, studies have found a positive correlation between elevated blood glucose and GGT levels in subjects with metabolic conditions (65, 66). Both markers are present at lower levels in *KCNJ2*-A allele carriers, strengthening the theory that the SNP may be reducing transduction and may have a protective effect on metabolic health.

In addition to the cross-sectional design of the study, the results require interpretation considering several limitations. As an elderly cohort, age-related decline in the perception of all five tastes is possible (18). Therefore, age adjustments were important in validating results for wider application. Age did not influence the relationships of statistical significance making findings applicable to broader population age ranges. Nutrient intake data were derived from an FFQ which can be subject to under and over-reporting, reporting bias and erroneous recall (67, 68). Furthermore, FFQs are more representative of habitual intake than specific daily intake (67). The findings are not necessarily causal in a cross-sectional study and in the absence of contextualising research, the hypotheses require further investigation.

The large sample size and even sex distribution of the study cohort are a strength of this study. While some patterns were found that suggest potential allele dose effects, it is important to note that the genotype analysis is provided for contextual patterns of allele dosage only, and limited statistical significance was found here, likely due to the reduced statistical power when analysing with three (AA/AG/GG) groups, as compared to two (presence/absence of the A-allele). Of those carrying the *KCNJ2*-A allele, 54.5% were female and therefore the sexes are evenly represented. In addition, the mean allelic frequencies are reflective of those found in European and Asian countries (69), cultures representative of the wider Australian populace (70). Importantly, the well-characterised study cohort enabled multiple outcome variables to be assessed and confounders to be adjusted for, improving the integrity of the findings.

## Conclusions

In presenting associations between *KCNJ2*-rs236514 and macronutrient, vitamin and mineral intakes, body composition, blood biomarkers of liver health and blood glucose levels, this novel research suggests the sour taste gene may be a modifier of nutritional intake and measures of metabolic health. Additional studies exploring the impact that the *KCNJ2*-rs236514 SNP has on sour detection thresholds, intensity and preference are required to clarify potential influence on dietary choice and intake. Understanding individual genetic taste profiles may then help health professionals customise diets that improve nutritional status and health. Further research is required on the effect the SNP may be having on signalling magnitude and direction to understand the mechanisms involved and test these hypotheses. In addition, investigating the possible extra-oral functions of the *KCNJ2* receptor and the rs236514 SNP may greatly assist in improving the health of those with overweight and obesity, liver disease and metabolic-related health conditions.

## Data Availability Statement

The raw data supporting the conclusions of this article will be made available by the authors, without undue reservation.

## Ethics Statement

The studies involving human participants were reviewed and approved by University of Newcastle Human Research Ethics Committee (Reference No. H-2008-0431). The patients/participants provided their written informed consent to participate in this study.

## Author Contributions

CF and EB: conceptualisation, formal analysis, methodology, and writing—original draught. CF, ML, and EB: data curation. MV, ML, and EB: funding acquisition, project administration, and resources. CF, AT, CS, MV, ML, TB, and EB: investigation and writing—review and editing. All authors have read and agreed to the published version of the manuscript.

## Conflict of Interest

The authors declare that the research was conducted in the absence of any commercial or financial relationships that could be construed as a potential conflict of interest.

## Publisher's Note

All claims expressed in this article are solely those of the authors and do not necessarily represent those of their affiliated organizations, or those of the publisher, the editors and the reviewers. Any product that may be evaluated in this article, or claim that may be made by its manufacturer, is not guaranteed or endorsed by the publisher.
